# Acetylation state of RelA modulated by epigenetic drugs prolongs survival and induces a neuroprotective effect on ALS murine model

**DOI:** 10.1038/s41598-018-30659-4

**Published:** 2018-08-27

**Authors:** Lorenzo Schiaffino, Roberta Bonafede, Ilaria Scambi, Edoardo Parrella, Marina Pizzi, Raffaella Mariotti

**Affiliations:** 1 Department of Neurosciences, Biomedicine and Movement Sciences, University of Verona, Verona, Italy; 2 Department of Molecular and Translational Medicine, University of Brescia, Brescia, Italy

## Abstract

Dysregulation in acetylation homeostasis has been implicated in the pathogenesis of the amyotrophic lateral sclerosis (ALS), a fatal neurodegenerative disorder. It is known that the acetylation of transcriptional factors regulates their activity. The acetylation state of NF-kB RelA has been found to dictate the neuroprotective versus the neurotoxic effect of p50/RelA. Here we showed that the pro-apoptotic acetylation mode of RelA, involving a general lysine deacetylation of the subunit with the exclusion of the lysine 310, is evident in the lumbar spinal cord of SOD1(G93A) mice, a murine model of ALS. The administration of the HDAC inhibitor MS-275 and the AMPK/sirtuin 1 activator resveratrol restored the normal RelA acetylation in SOD1(G93A) mice. The SOD1(G93A) mice displayed a 3 weeks delay of the disease onset, associated with improvement of motor performance, and 2 weeks increase of lifespan. The epigenetic treatment rescued the lumbar motor neurons affected in SOD1(G93A) mice, accompanied by increased levels of protein products of NF-kB-target genes, Bcl-xL and brain-derived neurotrophic factor. In conclusion, we here demonstrate that MS-275 and resveratrol restore the acetylation state of RelA in the spinal cord, delaying the onset and increasing the lifespan of SOD1(G93A) mice.

## Introduction

Amyotrophic lateral sclerosis (ALS) is a fatal neurodegenerative disease that affects upper and lower motor neurons (MNs). The MNs degeneration causes weakness, muscle atrophy and progressive paralysis of voluntary muscles, leading to a premature death usually due to respiratory failure. The 90% of ALS cases are sporadic, while the remnant 10% are familial and 20% of these are caused by the mutation in Superoxide Dismutase 1 gene (SOD1)^1^. The degeneration of MNs appears to be caused by the interaction of many factors: glutamate excitotoxicity, mitochondrial dysfunction, inflammatory response, impairment of axonal transport, oxidative stress and transcriptional dysregulation. In these regards, the ALS is considered a multifactorial disease^2^. To date, the use of drugs, alone or in combination, have been assessed to counteract two or more ALS degenerative mechanisms both in animal models^3,4^ and in patients^5^, without leading to real improvements.

Epigenetic drugs, modulating the enzymatic activity of histone deacetylases (HDACs) and histones acetyltransferases (HATs), have emerged as a potential tool to cure neurodegenerative diseases^6^, including ALS^7^. An unbalance of HATs and HDACs activity has been found in ALS^8,9^. The HDACs and HATs regulate the acetylation of histone proteins in the chromatin structure. The acetylation state of histones influences the transcriptional activity of the DNA. Furthermore, HDACs and HATs can modulate the acetylation of non-histones proteins like *nuclear factor kappa-light-chain enhancer of activated B cells* (NF-kB)^10^. NF-kB is formed by two of five DNA-binding proteins (p50, p52, p65 RelA, c-Rel, RelB) and the composition is essential to define its transcriptional activity^11^. It is known that the p50/RelA dimer can have a neuroprotective or neurotoxic effect depending on the acetylation state of RelA^12^. Preclinical studies on brain ischemia showed a pro-apoptotic activity of RelA associated with reduced global acetylation and an aberrant increase of acetylation at the lysine 310 (K310) residue^13^. Recently it has been reported that RelA subunit is increased in mutant SOD1 MNs in *in vitro* model of ALS and in spinal MNs of ALS patients^14,15^, supporting a direct correlation between RelA activation and MNs degenerations. Noteworthy, although those studies did not focus the RelA acetylation state in MNs, they reported that the MNs vulnerability to the mutated SOD1 astrocyte-conditioned medium was dependent on the activation of the phosphorylated form of RelA, known to enhance RelA acetylation at the K310 residue^16^. Here we demonstrated that pro-apoptotic acetylation state of RelA, encompassing global lysine deacetylation but enhanced K310 acetylation, was evident in the lumbar spinal cord of SOD1(G93A) mice, a murine model of ALS. On the bases of a synergistic neuroprotective activity displayed by a combination of the HDAC inhibitor MS-275 and resveratrol in brain ischemia^13^, by way of normalizing the RelA acetylation state, we tested the efficacy of the epigenetic treatment in the SOD1(G93A).

MS-275 is a synthetic benzamide inhibitor of HDACs 1-3^17,18^, that leads to an increase of the acetylation of histones proteins (e.g. histone 3, H3), is known to show an anti-tumor activity and is undergoing clinical trials for cancer treatment^19^. MS-275, through its inhibitory action, enhances the general RelA acetylation on the lysine residues^20^. Resveratrol is a natural polyphenol widely investigated for its anti-inflammatory, anti-oxidative, anti-proliferative and chemo-preventive properties^21^. This molecule is able to enhance the activity of the class III HDAC sirtuin 1 and the serine-threonine kinase AMP-activated kinase (AMPK), two enzymes involved in the modulation of RelA acetylation^13^. Our results demonstrate that the combined administration of these epigenetic drugs, tested at two different doses, both in the micrograms range, reestablished the proper acetylation state of RelA in the lumbar spinal cord of SOD1(G93A) mice. Most relevant, it provided a neuroprotective effect by causing a delay of the disease onset with an improvement of the motor performance and, finally, an elongation of animal survival.

## Results

### MS-275 and resveratrol enhance motor performance and increase survival of SOD1(G93A) mice

**Figure 1:**
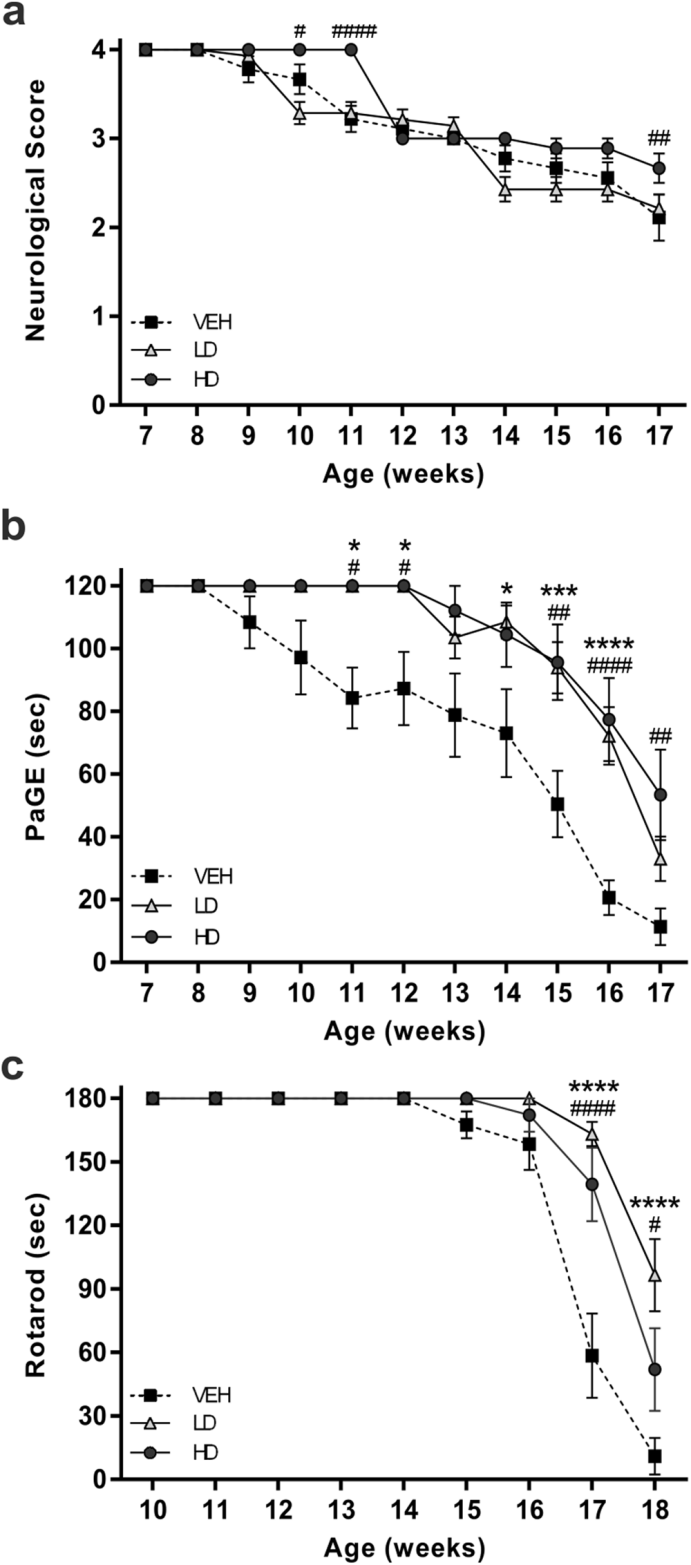
Motor performances of SOD1(G93A) mice. The graphs show the motor performances of SOD1(G93A) mice treated with vehicle (VEH) or with the pharmacological combination of resveratrol and MS-275 at low or high doses (LD and HD, respectively). (**a**) Neurological Score test shows statistically significant differences comparing HD (black circle) with VEH (black square) groups (VEH vs HD, ^##^P = 0.0153). (**b**) PaGE test shows a significant improvement of motor performance of LD (white triangle) and HD groups comparing to VEH mice (VEH vs LD, ^***^P = 0.0007; VEH vs HD ^##^P = 0.004). (**c**) Rotarod test shows a significant improvement of motor coordination of LD and HD groups starting from the 15th week. Note that the VEH group showed a faster decline of performances (VEH vs LD, ^***^P = 0.0006; VEH vs HD ^#^P = 0.0108). Results were analyzed by two-way ANOVA followed by Bonferroni multiple comparisons test. ^*^ was used to indicate differences between VEH and LD groups, while ^#^ was used to indicate differences between VEH and HD groups. Data in all graphs are expressed as mean ± SEM.

In order to evaluate the effect of MS-275 and resveratrol in SOD1(G93A) mice, behavioral tests were performed in all groups of animals: control group (VEH n = 10), Low Doses group (68 µg/Kg of resveratrol and 2 µg/Kg of MS-275; LD n = 14) and High Doses group (136 µg/Kg of resveratrol and 4 µg/Kg of MS-275; HD n = 10). The intraperitoneal administration of resveratrol and MS-275 improved motor performance of the treated animals when compared to control group (Fig. 1). Specifically, while no difference was detected in the Neurological Score between VEH and LD groups (VEH vs LD), a statistically different score was observed by increasing the drug doses (HD) (VEH vs HD, ^##^P = 0.0148). The HD group showed a significant delayed of Neurological Score deficit at 11 (^##^P = 0.0023) and 17 (^##^P = 0.004) weeks (Fig. 1a).

The grip strength of the animals was monitored with PaGE test^22^. The animals of the VEH group started to fail the test at the beginning of the 9^th^ week (108.4 ± 8.3 sec) and progressively declined their performances in the following weeks. The drug treatment significantly delayed the loss of motor function. Either the treated groups (LD, HD) showed a motor impairment at the 13^th^ week (LD, 103.5 ± 6.6 sec; HD 112.2 ± 7.7 sec) (Fig. 1b). Moreover, the decline of PaGE performances in LD and HD mice was significantly slower than in vehicle treated group (VEH vs LD, ^***^P = 0.0007; VEH vs HD ^##^P = 0.004).

When tested by the Rotarod test, the VEH group was the first to fail the task, by the 15th week (167.5 ± 6.3 sec). In addition, the VEH group showed a faster ongoing decline in the test performances comparing to both the treated groups, as shown in Fig. 1c (VEH vs LD, ^***^P = 0.0005; VEH vs HD ^#^P = 0.0055).

**Figure 2:**
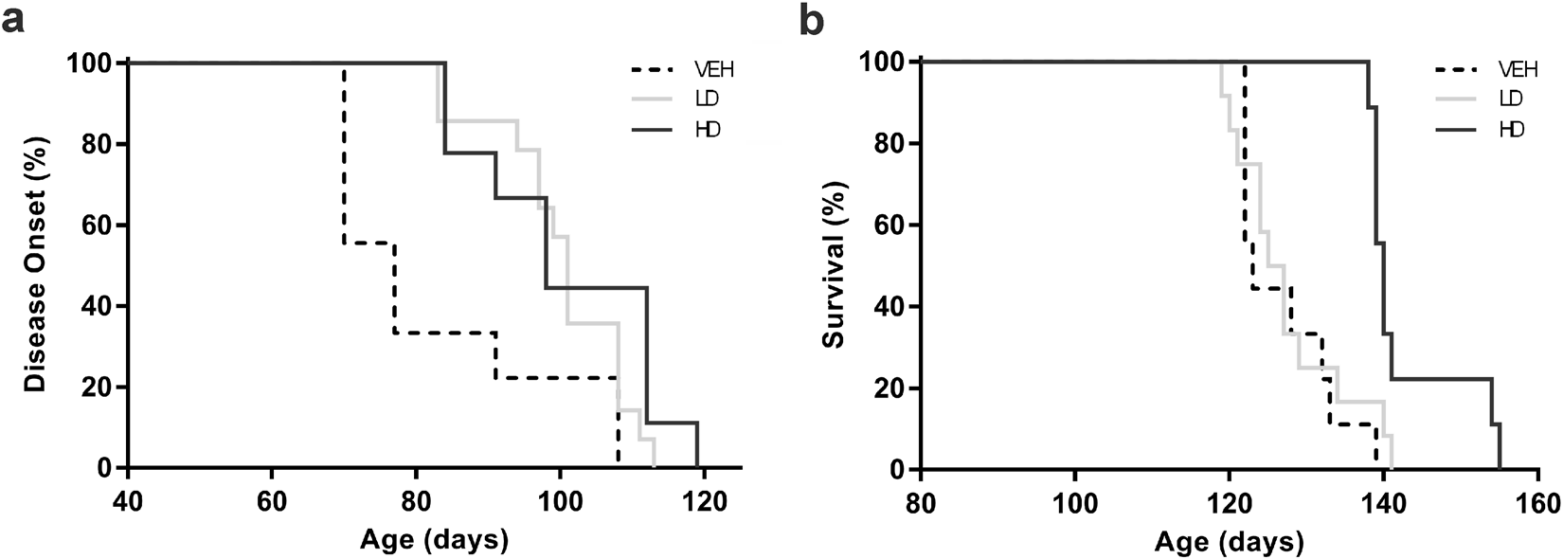
Disease onset and survival rate of SOD1(G93A) mice. The graphs show the disease onset and survival rate of SOD1(G93A) mice treated with vehicle (VEH) or with the pharmacological combination of resveratrol and MS-275 at low or high doses (LD and HD, respectively). (**a**) The graph shows the disease onset of the animals. The onset of LD (light grey line) and HD (grey line) mice was significantly delayed compared to VEH (black dotted line) animals. (**b**) The survival rate graph shows a statistically significant increase in lifespan of HD group comparing to VEH mice (VEH vs HD, ^###^P = 0.0004). Statistical analysis was performed with Log-rank (Mantel-Cox) test. Graphs show the percentages of occurrence of the events.

The onset of the disease appeared delayed by more than three weeks of mean in the drug treated groups when compared to vehicle treated animals (Fig. 2a). The disease onset of VEH group occurred at 81 ± 5 days, while in LD and HD groups it became detectable at 100 ± 2.4 days and 101 ± 4.3 days respectively (VEH vs LD, ^*^P = 0.0393; VEH vs HD ^#^P = 0.0122). Finally, the lifespan of SOD1(G93A) mice treated with vehicle (VEH group) was 126 ± 2.2 days. Despite no significant improvement of lifespan was produced by low dose treatment (Fig. 2b, VEH vs LD), the higher dose administration (HD group) significantly increased the mice survival by more than two weeks, corresponding to an increase of 12% compared to VEH (Fig. 2b, 143 ± 2.2 days, VEH vs HD, ^###^P = 0.0004).

### MS-275 and resveratrol protect lumbar spinal cord MNs from neurodegeneration

**Figure 3:**
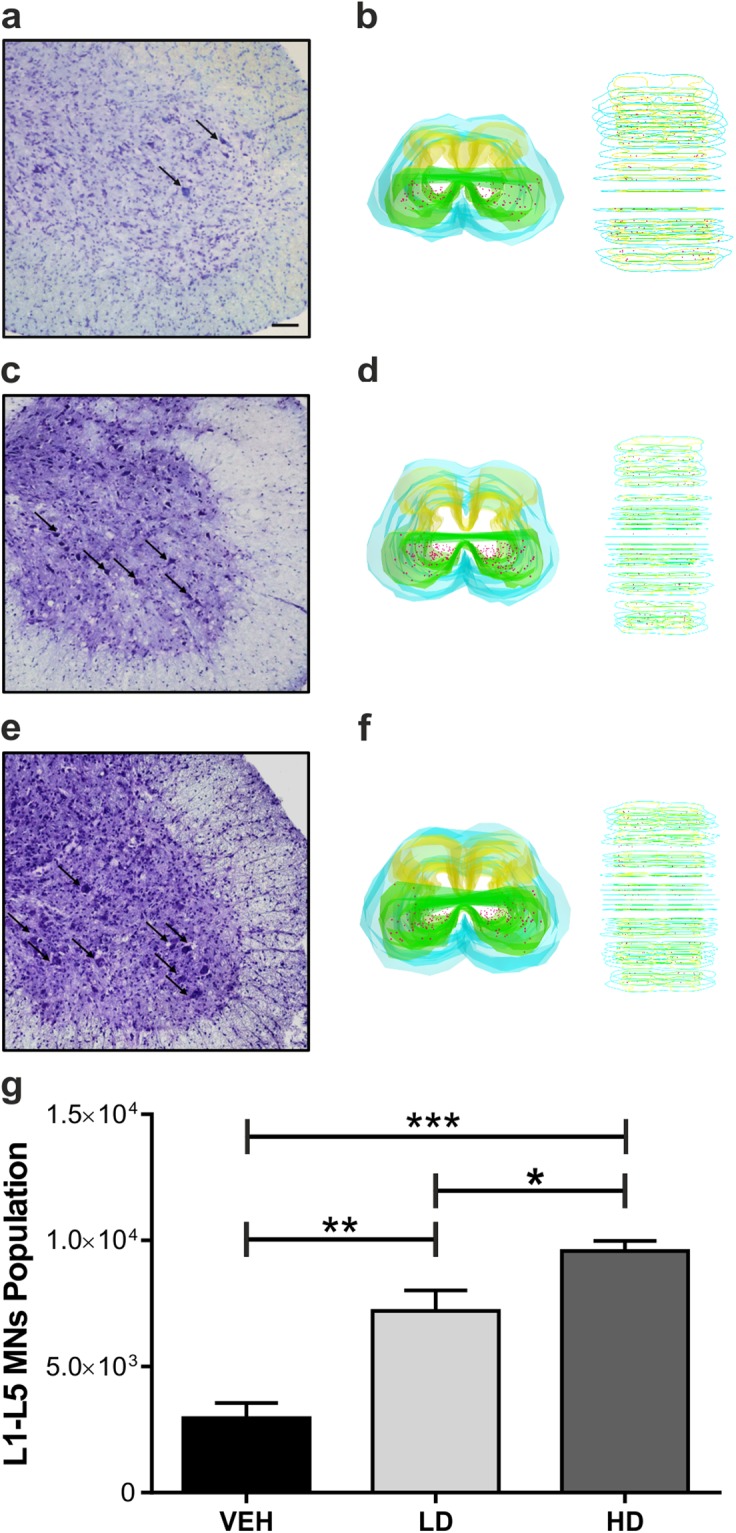
Number of MNs of the lumbar spinal cord of SOD1(G93A) mice. Treatment with epigenetic drugs increases the MNs survival in the lumbar spinal cord (L1-L5). (**a–c**) Nissl staining on the L5 segment of the spinal cord of VEH (n = 5) (**a**), LD (n = 5) (**b**) and HD (n = 5) (**c**) shows an increase of the numbers of MNs (arrows) in LD and HD mice compared to VEH. (**d**) 3D reconstruction of VEH lumbar spinal cord. (**e**) The graph shows the number of MNs population of the L1-L5 tract of the spinal cord of animal groups. Note a significant augment of the number of MNs population in a dose-dependent manner. Magnification 20×, scale bar 100 μm. Results were analyzed by one-way ANOVA followed by Tukey**’**s multiple comparisons test. Data are expressed as mean ± SEM. ^*^p < 0.05, ^**^ p < 0.01, ^***^p < 0.001.

To evaluate the neuroprotective effect of the epigenetic drug’s association, the stereological count was performed on ventral horn MNs population of lumbar spinal cord (L1-L5) at end stage in all experimental groups (Fig. 3a–f). The drugs protected the MNs from death in a dose-dependent manner. In particular, a significant increase of MNs number were observed in the spinal cord of SOD1(G93A) treated with low dose (n = 5) (7105 ± 455.3) compared with VEH group (n = 5) (2959 ± 486) (VEH vs LD, ^**^P = 0.0034) (Fig. 3g). Moreover, by increasing the drug dose we observed a dose-dependent neuroprotective effect. In this regard, the number of lumbar MNs of HD group (n = 5) (9665 ± 502.2) was significantly increased compared to VEH and LD groups (VEH vs HD, ^###^P = 0.0007; LD vs HD, ^#^P = 0.0129) (Fig. 3g). Moreover, immunohistochemistry analysis for IBA-1 on the lumbar spinal cord of WT (n = 3), VEH (n = 3) and HD (n = 3) mice was assessed to evaluate the effect of the treatment on the microglial activation. Despite a slight decrease of the positive microglial cells were detected in HD compared to VEH groups, significant statistical differences were not found (Supplemental Fig. 1).

### RelA acetylation state in lumbar spinal cord of SOD1(G93A) mice

**Figure 4:**
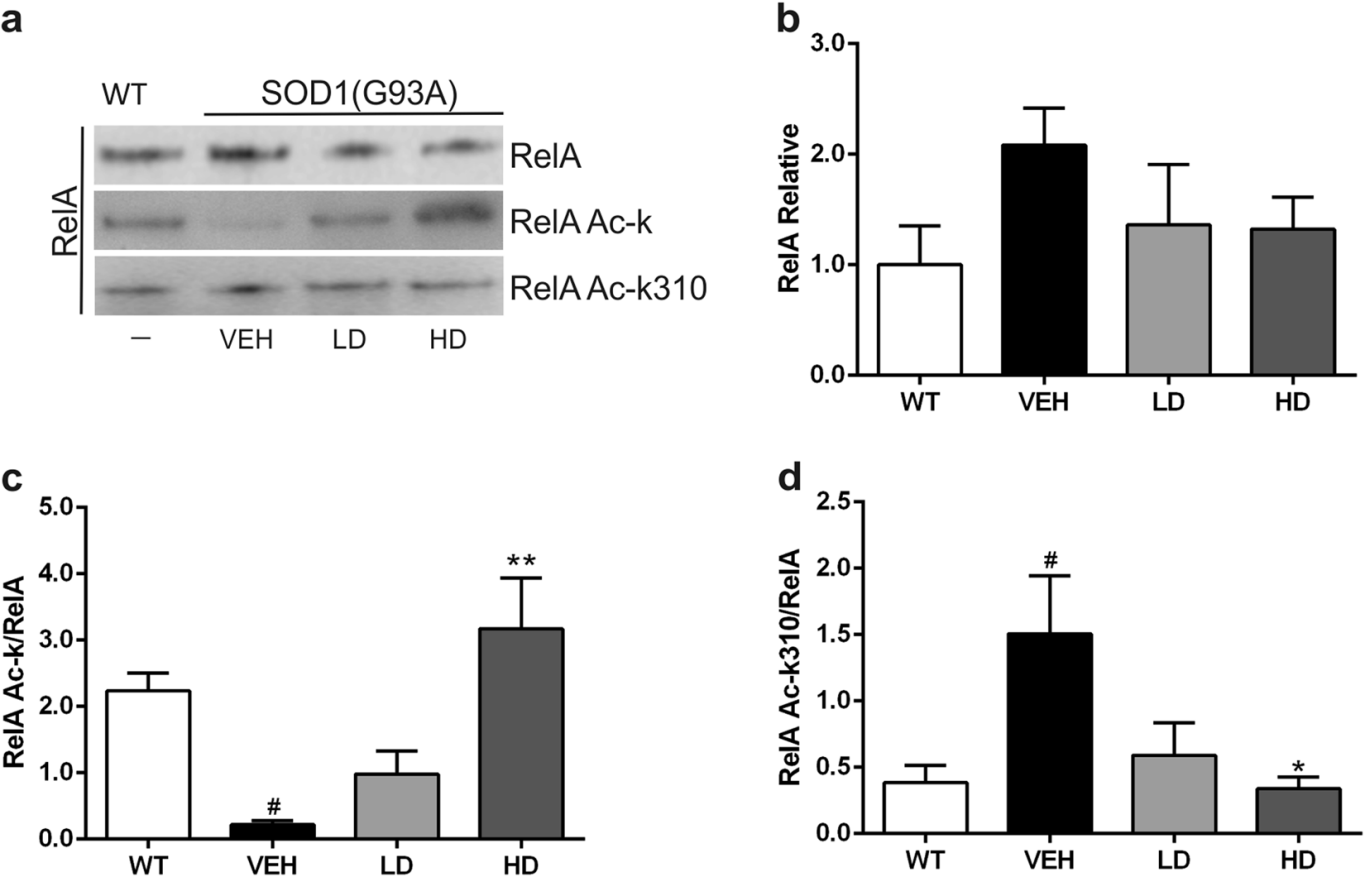
Immunoprecipitation and densitometric analysis of RelA subunit. Acetylation of RelA protein in the lumbar spinal cord of WT and VEH, LD and HD SOD1(G93A) mice. (**a**) Representative picture of the co-immunoprecipitation analysis of RelA acetylation in nuclear proteins extracted from lumbar spinal cord tissues. (**b**) Densitometric analysis of immunoprecipitated RelA. Despite a slight increase of the RelA expression in VEH group, no statistical differences were detected between the groups (**c**) Graph showing the amount of total RelA acetylated in relation to the RelA immunoprecipitated. The RelA acetylation was statistically reduced in VEH group compared to WT (WT vs VEH, ^#^P = 0.0324) while the treatment with the drugs at high doses significantly increased the acetylation of RelA (VEH vs HD, ^**^P = 0.0024). (**d**) The graph shows the acetylation of K310 in relation to the RelA immunoprecipitated. The acetylation at K310 of RelA was increased in VEH group compared to WT (WT vs VEH, ^#^P = 0.0446). The treatment decreases the RelA K310 acetylation in HD group compared to VEH (VEH vs HD, ^*^P = 0.036). All gels and blots were processed in parallel showing similar results. All the graphs show the densitometry analysis of immunoblot bands expressed as mean ± SEM. All data are expressed as the percentage of WT corresponding values. Results were analyzed by one-way ANOVA followed by Tukey**’**s multiple comparisons test. ^#^ indicates p < 0.05 of the interested group vs WT group; ^*^ and ^**^ indicate p < 0.05 and p < 0.01 respectively of the interested group vs VEH group. Full length blots are shown in Supplementary Information, in Supplemental Fig. 1.

After co-immunoprecipitation of RelA from the nuclear proteins fraction of the lumbar spinal cord, the acetylation state of RelA was evaluated on WT (n = 4) and at end stage on SOD1(G93A) (LD n = 4; HD n = 4; VEH n = 4) mice (Fig. 4a). A slight increase of the RelA expression in VEH group was detected, despite no statistical differences were reached (Fig. 4b). In the VEH group a general reduction of the acetylation state of RelA compared to WT mice (WT vs VEH, ^#^P = 0.0324) (Fig. 4c). Despite this significant reduction of the overall acetylation state of RelA, we found that the K310 residue of the RelA subunit resulted strongly acetylated compared to WT mice (WT vs VEH, ^#^P = 0.0446) (Fig. 4d). These results demonstrated that there is an alteration of the acetylation state of RelA in SOD1(G93A) mice. The acetylation state of the RelA subunit was re-established to the WT condition in HD group. However, LD group showed a slight increase of the total RelA acetylation and a decrease of the K310 acetylation, although no statistical differences were found (VEH vs LD) (Fig. 4c,d). Concerning the group treated with the highest doses, a significant increase of general RelA acetylation was detected (VEH vs HD, ^**^P = 0.0024), while the acetylation of K310 was statistically decreased compared to untreated mice (VEH vs HD, ^*^P = 0.036) (Fig. 4c,d).

### H3 acetylation state in the lumbar spinal cord of SOD1(G93A) mice

**Figure 5:**
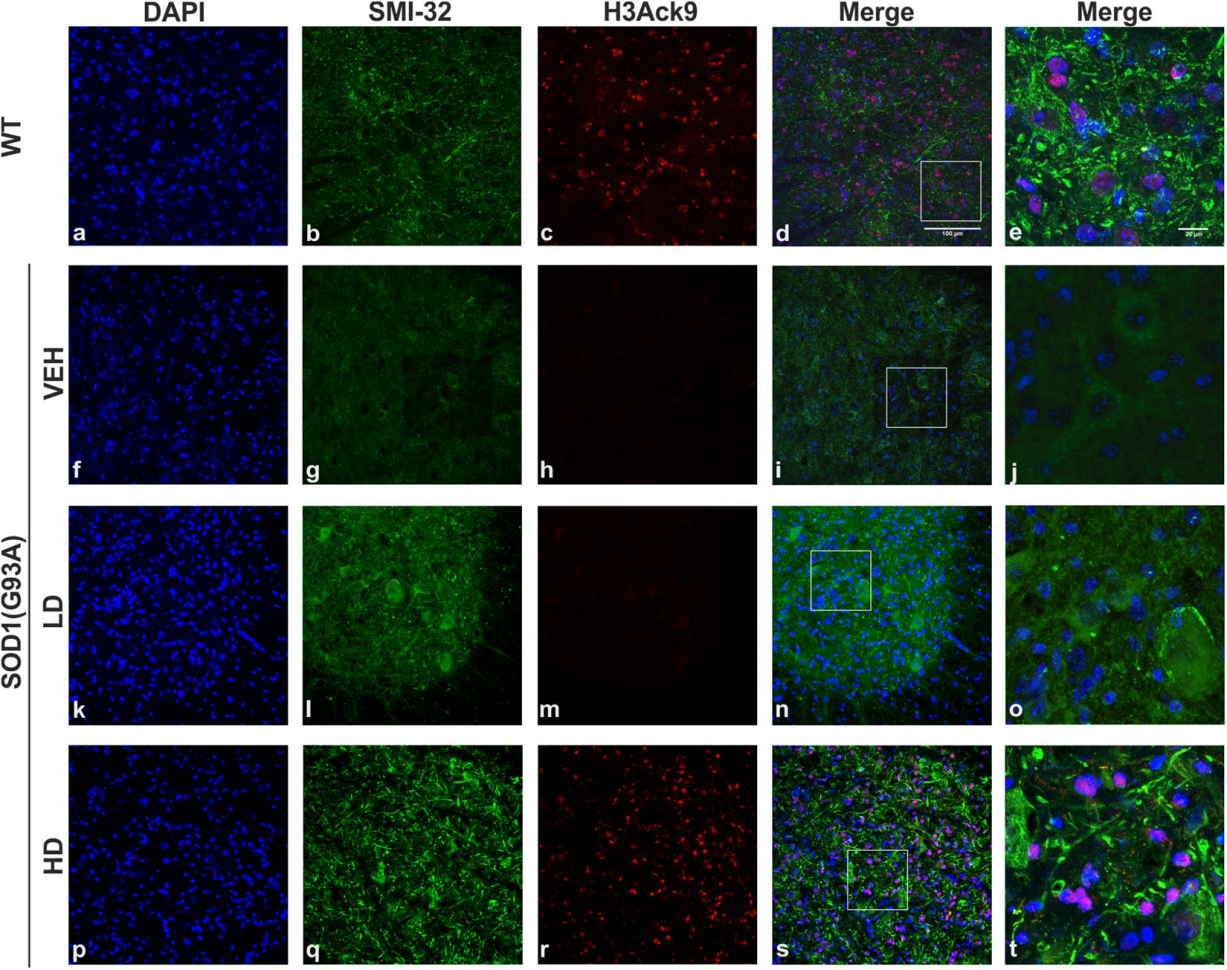
Histone 3 acetylation in the lumbar spinal cord of WT and SOD1(G93A) mice. The figure panel shows the different acetylation state of lysine 9 of histone 3 in the lumbar spinal cord of WT mice and VEH, LD and HD SOD1(G93A) groups (n = 4 per groups). The nuclei were stained in blue with DAPI (**a**,**f**,**k**,**p**) while, to identify MNs, the antibody neurofilament H was detected with SMI-32 antibody in green (**b**,**g**,**l**,**q**). The acetylation of histone 3, identified by H3Ack9 antibody in red was not present in VEH (**h**) and LD (**m**) groups compared to WT animals. The treatment with the epigenetic drugs restores the acetylation of histone 3 in HD group. Magnification 20×, scale bar 100 µm (**a–d**, **f–i**, **k–n** and **p–s**). In the last column (**e**,**j**,**o**,**t**) are shown the higher magnification of the boxed area showed in d, i, n and s respectively, scale bar 20 µm.

In order to evaluate the effect of the treatment, in particular the inhibition of HDACs class I induced by MS-275, we investigated the acetylation of H3 in the lumbar spinal cord of WT, VEH, LD and HD groups. We examined the acetylation of the Lys 9 of H3 (H3Ack9) (Fig. 5c,h,m,r) in the nuclei (DAPI) (Fig. 5a,f,k,p) of lumbar spinal cord MNs (SMI-32) (Fig. 5b,g,l,q) of SOD1(G93A) end stage (VEH, LD and HD) and littermate control mice (WT) (Fig. 5). The qualitative examination of the immunohistochemistry images showed a drastically decreased of the acetylation of H3 in VEH group (Fig. 5f–j) compared to WT group (Fig. 5a–e), confirming data already reported in different *in vivo* models of ALS^23^. We found that the administration of low doses of the drugs in SOD1(G93A) did not induce an increase of the acetylation of H3 (Fig. 5k–o), demonstrating that the administration of 2 µg/Kg dose of MS-275 did not modulate the acetylation state of H3. Interestingly, the dose of MS-275 (4 µg/Kg) used in HD group determined an increase of the acetylation of H3 of SOD1(G93A) mice (Fig. 5p–t) compared to VEH group (Fig. 5f–j). Our results showed that the dose of MS-275 used in HD group is able to modulate the activity of HDACs class I in our mice model, restoring the correct acetylation homeostasis.

### Drugs treatment increase the phosphorylation of Thr172 of AMPK in the lumbar spinal cord of SOD1(G93A) mice

**Figure 6:**
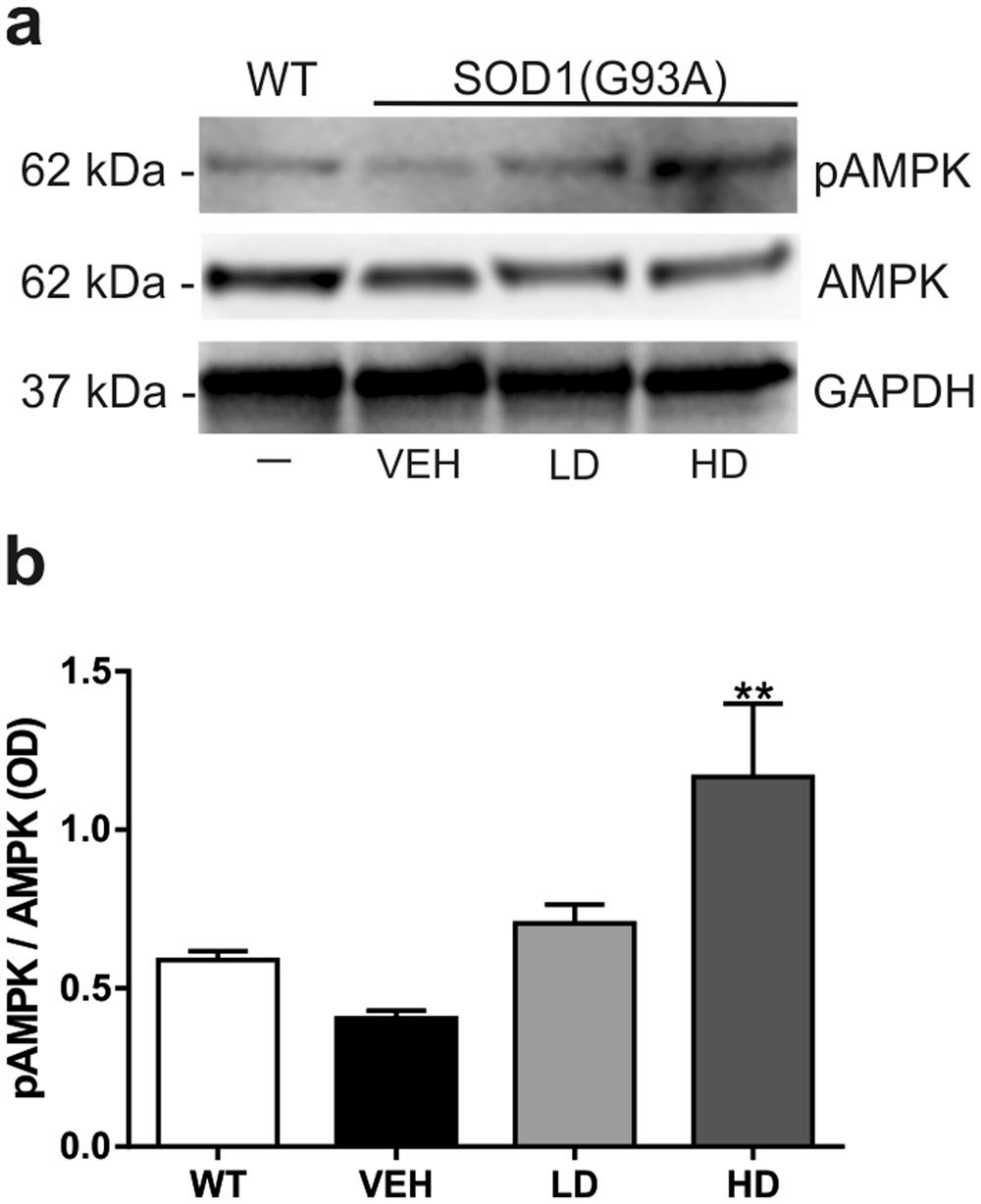
Analysis of phosphorylation of AMPK. Western blot assay was performed in the cytoplasmic fraction of the lumbar spinal cord of WT mice and VEH, LD and HD SOD1(G93A) groups (n = 3 per group). (**a**) Representative western blot image showing the phosphorylation rate pAMPK (62 kDa) and the expression of AMPK (62 kDa) and GAPDH (37 kDa). (**b**) The graph shows the densitometric analysis of the phosphorylation of pAMPK compared to the total expression of AMPK. The VEH show a slight decrease (not significant) of the phosphorylation of AMPK compare to WT. The treatment increase the pAMPK/AMPK ratio and significant differences were found between VEH and HD group (VEH vs HD, ^**^P = 0.089). All gels and blots were processed in parallel showing similar results. Results were analyzed by one-way ANOVA followed by Tukey**’**s multiple comparisons test. Graphs are shown as mean ± SEM. ^*^ and ^**^ indicate p < 0.05 and p < 0.01 respectively of interested group vs VEH group. Full length blots are shown in Supplementary Information, in Supplemental Fig. 2.

In order to examine the effect of resveratrol on the protein target AMPK, we analyzed the phosphorylation rate of Thr172 of AMPK (pAMPK) in the cytoplasmic protein fraction of lumbar spinal cord of end stage VEH, LD and HD groups and littermate control WT mice. We detected a slight decrease of the phosphorylation rate of Thr172 of pAMPK in VEH compared to WT (no statistical differences) (Fig. 6a). This data confirmed the results reported in a previous study^24^. Moreover, we found that the drug administration increased the phosphorylation of AMPK in LD, restoring the phosphorylation at the level similar to WT group (Fig. 6b). In HD group a significant difference was found compared to VEH (VEH vs HD ^**^P = 0.0089).

### MS-275 and resveratrol association increased the neurotrophic factor BDNF and anti-apoptotic Bcl-xL protein levels in lumbar spinal cord

**Figure 7:**
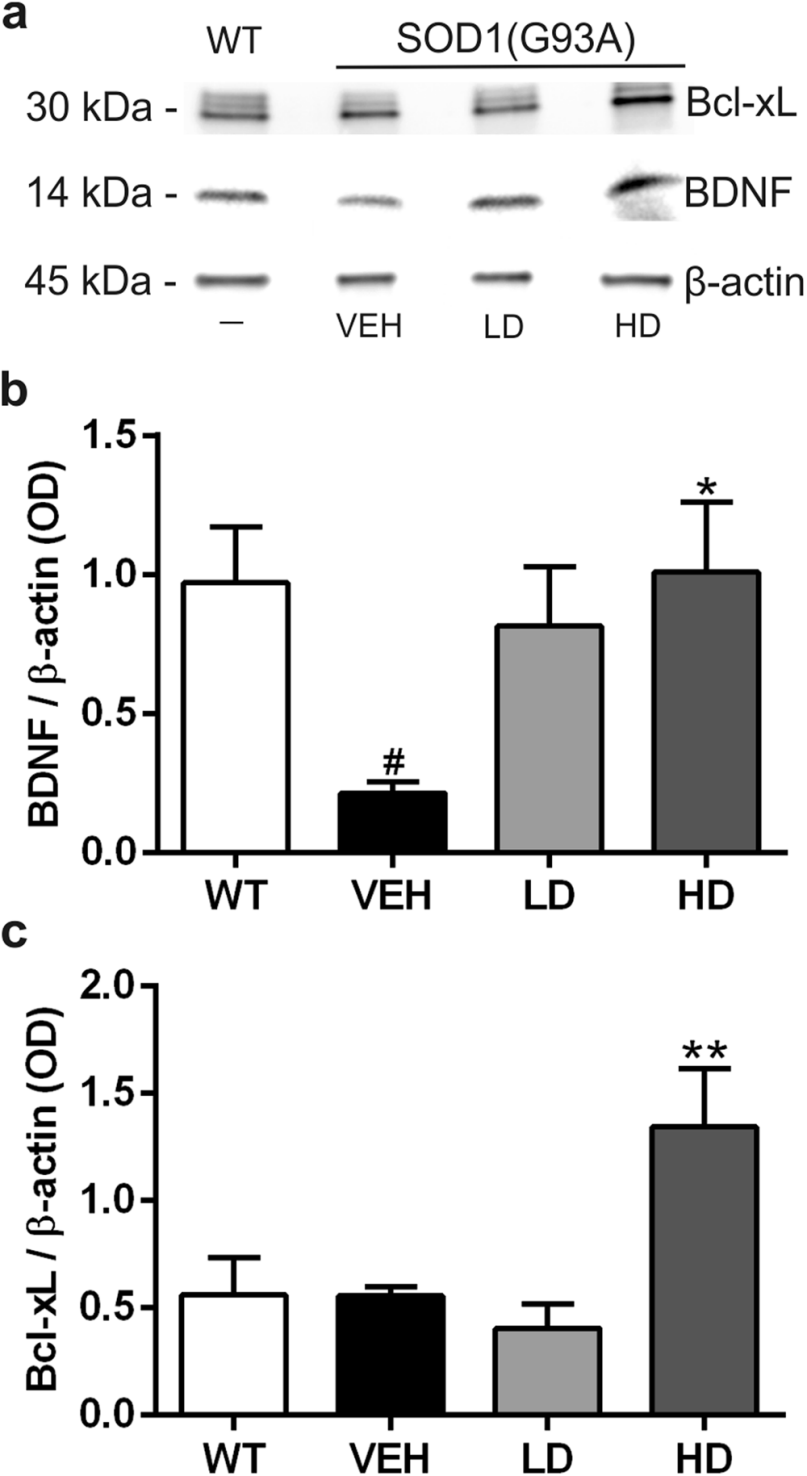
Analysis of neurotrophic (BDNF) and anti-apoptotic (Bcl-xL) factors expression levels. Western blot assay was performed in the cytoplasmic fraction of the lumbar spinal cord of WT mice and VEH, LD and HD SOD1(G93A) groups (n = 5 per group). (**a**) Representative western blot image depicting the expression of BDNF (14 kDa), Bcl-xL (30 kDa) and β-actin (45 kDa). (**b**–**c**) Densitometric analysis of the expression of BDNF and Bcl-xL normalized to β-actin. In (**b**) note the significantly decreased expression of BDNF in VEH group compared to WT (WT vs VEH, ^#^P = 0.0280) and the increase of BDNF expression after drugs treatment, statistically significant in HD group (VEH vs HD, ^*^P = 0.0206). In (**c**) note the significant increase of Bcl-xL expression after the treatment with the high dose of drugs (VEH vs HD ^**^P = 0.0054). All gels and blots were processed in parallel showing similar results. Results were analyzed by one-way ANOVA followed by Tukey**’**s multiple comparisons test. Graphs are shown as mean ± SEM. ^#^indicates p < 0.05 of interested group vs WT group; ^*^ and ^**^indicate p < 0.05 and p < 0.01 respectively of the interested group vs VEH group. Full length blots are shown in Supplementary Information, in Supplemental Fig. 3.

The cytoplasmic protein fraction of lumbar spinal cord of WT (n = 5), VEH (n = 5), LD (n = 5) and HD (n = 5) was analyzed at end stage for the detection of brain-derived neurotrophic factor (BDNF) and Bcl-xL protein expression levels (Fig. 7a). We found that the neurotrophic factor BDNF was constitutively expressed in WT lumbar spinal cord, while it was downregulated in SOD1(G93A) control mice (WT vs VEH, ^#^P = 0.0280) (Fig. 7b). The drug administration increased the BDNF levels in both LD and HD treated groups and significant differences were found comparing the animals treated with high doses to control condition (VEH vs HD, ^*^P = 0.0206) (Fig. 7b). The administration of MS-275 and resveratrol promoted the expression of the anti-apoptotic Bcl-xL protein in the lumbar spinal cord. Densitometric analysis showed that Bcl-xL levels in HD mice were significantly increased compared to VEH animals (VEH vs HD ^**^P = 0.0054) (Fig. 7c).

## Discussion

The causes of the neurodegenerative processes that lead to the death of MNs in ALS are still debated and unclear. It is known that ALS is a multifactorial disease, caused by interaction between genes, environmental factors, and altered molecular pathways^2^. Moreover, transcriptional gene dysregulation has been demonstrated to be a key factor in ALS, contributing to the progressive loss of MNs^25^. This gene dysregulation reflects an unbalanced DNA acetylation that leads to the transcription of factors, triggering the neurodegenerative processes^26^. The modulation of the enzymatic activity of HDACs and HATs leads to a homeostatic level of acetylation^6^. In literature, several *in vivo* studies on ALS models have been addressed to ameliorate the disease course, restoring a proper acetylation balance in MNs through the modulation of HDACs. Rouaux and colleagues demonstrated that sodium valproate (250 mg/kg/day), an inhibitor of HDACs, slightly prevented MNs degeneration and improved motor functions in the SOD1(G86R) mouse model of ALS, showing a delay of onset of 10% compared to untreated once but without improving mean survival^25^. Sodium valproate was tested in an ALS patients clinical trial, without any benefit^27^. Treatment of SOD1(G93A) mice with trichostatin A (1 mg/kg/days), another HDACs inhibitor, attenuates MNs loss, reduced gliosis, muscular atrophy and neuromuscular junction denervation. Moreover, this treatment was found to improve motor performances and promote an increase of only 7% in the lifespan of SOD1(G93A) compared to vehicle^28^. In addition, sodium phenylbutyrate (400 mg/kg/day) was documented to regulate the expression of anti-apoptotic genes ameliorating motor function and prolonging the survival of SOD1(G93A) mice of 21% compared to control^7^. Recent studies also showed that resveratrol, enhancing the enzymatic activity of SIRT1, exerted *per se* a neuroprotective effect on MNs and on muscular fibers^24,29^. In particular, Song and colleagues found that the administration of resveratrol (25 mg/kg/day) increased the lifespan of SOD1(G93A) mice of 11% compared to controls. The administration of HDAC inhibitor or resveratrol has been reported to increase the lifespan of ALS murine models. However, in these studies, a very high concentration of both drugs was used compared to our experimental doses. In our study, using resveratrol 200-fold less concentrated than the one used by Song and colleagues^29^ and MS-275 50-fold lower than that eliciting neuroprotection in models of brain ischemia^30^, we found a delay of 20 days of disease onset and a 12% of increase of lifespan of treated SOD1(G93A) mice. In our study, the better outcomes were achieved with the higher doses of the drugs combination (136 µg/kg of resveratrol per day and 4 µg/kg of MS-275 per day). All these data showed that the modulation of the enzymatic activity of HDAC by single drugs requires a very high concentration compared to that used in our study. The low drug doses can minimize possible side effects and the combination of MS-275 and resveratrol seemed to modulate better the neuroprotective effect on ALS mice model and appears appealing for possible clinical applications in ALS treatment.

It is known that the HDACs deacetylate histones and also transcriptional factors, including NF-kB^31^. It has been reported that the altered acetylation of NF-kB RelA subunit, i.e. a global reduction of acetylation state combined with a site-specific acetylation at the K310 residue, triggers the transcription of pro-apoptotic factors inducing neurodegeneration^13,32^. A body of evidence suggests a possible pathogenic role of NF-kB in ALS^33–35^. Overexpression of RelA subunit has been reported in spinal MNs from patients affected by ALS^14,36^. Notably, Ikiz and colleagues recently demonstrated an involvement of the RelA subunit in MNs degeneration in *in vitro* models of ALS^15^. On the basis of these findings, we investigated whether MNs death was associated with altered RelA acetylation state in the SOD1(G93A) murine model of ALS. Here we demonstrate, for the first time, an aberrant acetylation of lysine residues of RelA subunit in the lumbar spinal cord of SOD1(G93A) mice, with a general reduction of RelA lysine residues acetylation and a specific increased of acetylation of K310 residue. With the aim of restoring the correct RelA acetylation and rescue MNs from death, we treated the SOD1(G93A) mice with the combination of MS-275, a molecule able to enhance the global lysine acetylation of RelA protein, and resveratrol, a sirtuin1 activator promoting the selective deacetylation of RelA on K310^13^. Our results demonstrated that the combined administration of resveratrol and MS-275 reverted the altered acetylation state of RelA in the lumbar spinal cord of the SOD1(G93A) mice. The beneficial effect of the treatment at neuropathological level was coupled to an improvement of the motor performances, an evident delay of the disease onset and a remarkable increase of the survival of SOD1(G93A) mice.

This epigenetic treatment showed to be effective in protecting lumbar spinal cord MNs of SOD1(G93A) mice as it rescued them from death. The neuroprotective effect was also associated with increased level of the anti-apoptotic protein Bcl-xL, a member of the Bcl-2 family target of NF-κB. The Bcl-xL protein was increased in HD group, in which we found a higher number of survived MNs. It has been reported that Bcl-xL protein exerts a neuroprotective effect on MNs and, by counteracting the apoptotic pathway, can extend the survival in SOD1(G93A) mice^37,38^. We recently demonstrated that MS-275 and resveratrol promote a particular acetylation state of RelA that allows NF-kB to bind Bcl-xL gene promoter and increase the acetylation of promoter associated H3 histones, a process leading to Bcl-xL expression^13^. In the present study, we confirmed that restoring of the proper acetylation state of RelA protein, through the modulation of the enzymatic activity of HDACs, enhanced the Bcl-xL protein level in the lumbar spinal cord of SOD1(G93A) mice.

Moreover, we found that the increased number of MNs in treated groups was accompanied by an increased BDNF expression in the lumbar spinal cord of SOD1(G93A) mice. The BNDF neurotrophic factor involved in the regulation of brain development, synaptic plasticity, and memory function^39^. Bemelmans and colleagues showed that the viral-mediated gene transfer of BDNF in *in vivo* model of excitotoxicity had a neuroprotective effect resulting in a reduction of the lesioned area^40^. Recently, it has been observed a role of BDNF in counteracting the neurodegenerative processes in *in vitro* model of ALS^41,42^. Concerning our study, we observed a downregulation of BDNF in the lumbar spinal cord of SOD1(G93A) mice and we demonstrated that the combined administration of the two epigenetic drugs promoted the expression of BDNF in SOD1(G93A). Many studies reported that SIRT1 is involved in the expression of BDNF^43–45^. Moreover, Zeng and Yang demonstrated that resveratrol directly acts on SIRT1 activation, inducing BDNF expression^45^. Our study confirmed that BDNF expression was correlated in a dose dependent manner of resveratrol administration.

Our data reveal that the effect of the treatment with low drug doses elicited a significant increase of MNs survival that was not paralleled by increased Bcl-xL level or elongated animal lifespan. We retain that such a discrepancy could be due to the weak synergistic effect of the administered drugs at those low doses. In fact, the concentration of 2 µg/Kg of MS-275 did not induce an increase of the RelA and H3 acetylation, showing that this low dose is not sufficient to solve comprehensively its function. Nevertheless, the resveratrol administered in the LD group (68 µg/Kg), is able to efficiently activate its target SIRT1, inducing a deacetylation of K310 RelA subunit and an increase of the BDNF expression. In this regard, we retain that the increased MNs survival in the LD group could be due to the effect of the action of resveratrol and not by the synergistic effect of both drugs, that alone is not able to induce an increase of expression of Bcl-xL. Notably, increasing the dose of MS-275 (4 µg/Kg) we showed an enhanced effect of the protein target, supported by a strong increase of RelA acetylation state and an augmented acetylation of H3 in the spinal cord of SOD1(G93A). The HD treatment seems to better modulate the expression of neuroprotective and pro-survival molecules, as Bcl-xL and BDNF, through a fine modulation of SIRT1 and the HDACs class I, as confirmed by the higher number of rescued MNs, the delayed disease onset and increased lifespan found in treated mice compared to controls.

In conclusion, our study demonstrates that the combined administration of MS-275 and resveratrol may represent a promising therapeutic approach to cure ALS. Future studies will be addressed to further elucidate the mechanism of action of this promising pharmacological strategy and to test other similar combinations of epigenetic drugs.

## Methods

### Animals

Experiments were performed using transgenic mice overexpressing human *SOD1* carrying a Gly93−Ala mutation (SOD1(G93A)) (strain designation: B6SJL−TgN[SOD1−G93A]1Gur, stock number 002726) (n = 34) and wild-type (WT) (B6SJL) (n = 8) obtained from Jackson Laboratories (Ben Harbor, ME, USA). The experiments were performed with the approval of the Italian Minister of Health, following the National Institute of Health (NIH) guide for the use and the care of laboratory animals, in accordance with the current European Communities Council Directive (2010/63/EEC) and conformity to the international guidelines^46^, minimizing the number of animals used and avoiding their sufferance. The mice were maintained under controlled environmental parameters with food and water *ad libitum*, with 12 hours of light and dark cycle. The genotype of newborn mice was identified by polymerase chain reaction (PCR) specific for human SOD1 gene. The primers for the human SOD1 gene were: Forward (113), 5′-CATCAGCCCTAATCCATCTGA-3′; Reverse (114) 5′-CGCGACTAACAATCAAAGTGA-3′; while for the housekeeping gene interleukin-2 receptor (IL-2R) were: Forward (42) 5′-CTAGGCCACAGAATTGAAAGATCT-3′; Reverse (43) 5′-GTAGGTGGAAATTCTAGCATCATCC-3′.

### Behavioral tests

In order to test the efficacy of the treatment, SOD1(G93A) mice were weekly evaluated blinded for body weight, Neurological Score test, Paw Grip Endurance test (PaGE) and Rotarod test starting at 40 days of life. Neurological Score test was evaluated as follow: 4 normal (no sign of motor dysfunction); 3 hind limbs tremors were present when the mice were suspended by tail; 2 gait abnormalities; 1 dragging at least one hind limb; 0 inability to right itself in 30 sec when animal was placed on the supine position. PaGE was used to assess the grip strength of animals: mice were placed on a metal grid and quickly turned over. Two attempts were given and 120 sec was used as cut-off time. The latency time, measured as the time until the animals detached the hind limbs, was registered. Rotarod test was used to assess the motor coordination of the mice. The animals were placed in a rotor tube (Acceler Rota-Rod 7650, UGO BASILE, Italy) at the constant speed of 16 rpm. The cut-off time was settled at 180 sec, three attempts were given to mice that failed the test, with a resting phase of 5 min. The longest latency time was registered.

The animals failed the PaGE or Rotarod test when they are not able to reach the cut-off time. The onset was established when the mouse failed in PaGE or Rotarod test. When Neurological Score was equal to 0 the animals were sacrificed and the survival time was recorded.

### Pharmacological treatment

MS-275 (Entinostat) (BPS-27011, Vinci Biochem, Italy) and resveratrol (554325, Merck Millipore) were dissolved in dimethyl sulfoxide (DMSO), diluted in PBS. The solution was daily prepared from the stock and injected intraperitoneally in SOD1(G93A) mice. The final DMSO concentration injected was 0.1%. The animals were treated from 50^th^ days of life until the sacrifice day. Resveratrol and MS-275 were administered at doses previously reported to elicit synergistic neuroprotection in brain ischemia and selected on the bases of *in vitro* potency and PK studies^13^. The SOD1(G93A) mice were divided in three balanced sex groups: Vehicle (VEH) group (n = 10) received vehicle and was used as control; Low Dose (LD) group (n = 14) received 68 µg/Kg of resveratrol and 2 µg/Kg of MS-275 per day; High Dose (HD) group (n = 10) received 136 µg/Kg of resveratrol and 4 µg/Kg of MS-275 per day. The doses chosen in this study were well-tolerated *in vivo* and showed neuroprotective activity in a mouse model of ischemic stroke^13^.

### Immunoprecipitation and western blot

The SOD1(G93A) and WT (age matched) mice were sacrificed by cervical dislocation and the lumbar spinal cord was rapidly dissected out. The nuclear and cytoplasm protein isolation was performed as described before^47^. Protein concentration was determined using the Pierce™ Detergent Compatible Bradford Assay (23236, Thermo Fisher Scientific). Immunoprecipitation and western blot assays of nuclear fraction were used to detect the RelA acetylation. The nuclear protein fraction (50 µg) was suspended in 220 µl of buffer used for protein extraction and incubated with 30 µl of beads (protein A Sepharose CL-4B, 10233478, GE Healthcare) for 30 min at 4 °C on a rotator plate. The beads were then pelleted for 5 min at 1000 g and supernatant was collected and incubated with goat anti-RelA (10 µg/mg lysate, ab176821, Abcam) at 4 °C overnight on a rotator plate. After incubation with antibody, 35 µl of beads were added to the solution and the sample was incubated for 2 hours at 4 °C on a rotator plate. To obtain the beads binding to the immunocomplex, the sample was centrifuged for 5 min at 1000 g and washed twice with RIPA buffer solution (Na_3_VO_4_ 1 mM, NaCl 140 mM, Tris-HCl pH 7.8 10 mM, Nonidet P-40 0.5%) and once with PBS. The immunocomplex was detached from beads by boiling in Laemli buffer and 2.5% beta-mercaptoethanol. The immunoprecipitated proteins were analyzed by 4–12% SDS-polyacrylamide gel (#3450123, Biorad) and blotted on a PVDF membrane incubated for 2 hours in blocking solution. The detection of the immunoprecipitated proteins was performed with the following antibodies: rabbit anti-NF-kB p65 (1:1000, GTX107678, GeneTex), rabbit anti-Acetyl Lysine (1:500, AB3879, Merck Millipore) and rabbit anti-NF-kB p65 (acetyl K310) (2.5 µg/ml, ab19870, Abcam). To detect the expression of anti-apoptotic and neurotrophic factors, the cytoplasmic lysates (30 µg) were analyzed by western blot, as described above, using the following antibodies: rabbit anti- Bcl-xL (1:1000, #2762, Cell Signaling), mouse anti-BDNF (1:1000, ab203573, Abcam) and mouse anti-β-actin (HRP coniugated) (1:1000, #12262, Cell Signaling). Concerning the detection of AMPK and P-AMPK proteins, the analysis by western blot were performed as described above using the following antibodies: murine AMPKα rabbit mAb (1:1000, #5831, Cell Signaling) and p-AMPKα (Thr172) rabbit mAb (1:1000, #2535, Cell Signaling) and a mouse monoclonal anti-GAPDH (1:2000, ThermoFisher Scientific, AM4300). Values are expressed as P-AMPK/AMPK optical density percent ratios. The membranes were probed with horseradish peroxidase (HRP) polyclonal goat anti-rabbit immunoglobulins (1:2000, #P0448, Dako) or HRP polyclonal anti-mouse (1:2000, #P016, Dako) for 1 h in BSA 5% and PBS-T 0,1%. The membranes were then incubated with a chemiluminescent HRP substrate (WBKLS0500, Merck Millipore) and detected with G:BOX F3 GeneSys (Syngene, UK).

### Stereological count of the lumbar spinal cord motor neurons

The end stage SOD1(G93A) mice (n = 5 per group) were deeply anesthetized and transcardially perfused with PBS 0.1 M followed by paraformaldehyde (PFA) 4%. The spinal cord was dissected out and post-fixed with PFA 4% overnight. The lumbar tract was soaked in 30% sucrose, included in OCT and serially cut at 20 µm with cryostat apparatus. The sections were mounted on Surgipath®Apex™ Superior Adhesive Slides (3800080E, Leica Biosystems).

For Nissl Staining the slides were air-dried and then hydrated with H_2_O for 30 sec. After the staining with 0.2% cresyl violet solution for 8 min, the sections were gradually placed into increasing concentrations of ethanol, cleared with xylene, mounted with Entelan and covered with cover glass. The MNs of the lumbar tract (lateral and medial motor columns of L1-L5 spinal cord segments) were counted blinded every 100 µm by the operator using a computer-assisted microscope (Olympus BX6 with Retiga 2000R camera) with the Stereoinvestigator software (MicroBrightField, Williston, VT, USA) at 40x magnification.

### Immunohistochemistry on the spinal cord

To investigate the acetylation state of the Lys 9 of H3, immunofluorescence for H3Ack9, SMI-32 and DAPI were performed on lumbar spinal cord MNs of end stage SOD1(G93A) and age-matched WT (120 days). The slides were incubated for 1 h in 2.5% of Normal Goat Serum (NGS) and 0.3% Triton X-100 in PBS. The slides were then incubated overnight in H3Ack9 (1:250, GeneTex, GTX88007) and SMI-32 (1:1000, Biolegend, #SMI-32P) antibodies in 1.25% NGS and 0.3% Triton X-100 in PBS. After rinsing, the sections were incubated for 1 h in goat anti-rabbit 594 IgG (1:1000, A11012, ThermoFisher Scientific) and goat anti-mouse IgG 488 secondary (1:1000, A32723, ThermoFisher Scientific) antibodies in 1% NGS and 0.3% Triton X-100 in PBS. The nuclei were counterstained with DAPI (4′,6-Diamidino-2-Phenylindole, Dihydrochloride) (1:2000, D1306 ThermoFisher Scientific) for 5 minutes. After washings with PBS, sections were coverslipped with Fluorescent Mounting Medium (S3025, Dako). Immunofluorescence was analyzed with a TCS-SP5 confocal microscope (Leica-Microsystems, Wetzlar, Germany), in a dual-channel acquisition setup, using UV, 488 nm, and 543 nm excitation beams.

To evaluate gliosis activation, immunohistochemistry for light microscopy was performed to detect microglia cells on the lumbar tract of the spinal cord. The sections were incubated for 15 minutes in 1% H_2_O_2_ to quench endogenous peroxidase and preincubated for 1 h in 5% of NGS and 0.3% Triton X-100 in PBS. The slides were then incubated overnight in mouse anti-mouse Iba1 antibody (1:500, GTX89367, Gene Tex) in 1% NGS and 0.3% Triton X-100 in PBS. After rinsing, the sections were incubated for 1 h in biotinylated horse anti-goat IgG (1:200, BA-9500, Vector Laboratories) in 1% NGS and 0.3% Triton X-100 in PBS. The reaction was developed with the avidin-biotin peroxidase kit (ABC kit; Vector) using 3–3′-diaminobenzidine as chromogen. After mounting on slides, the sections were dehydrated through increasing grades of ethanol, cleared in xylene, and coverslipped with Entellan (Merck, Darmstadt, Germany). The microglial cells of the lumbar tract (L1-L5) were visualized using a computer-assisted microscope (Olympus BX6 with Retiga 2000R camera) with the Stereoinvestigator software (MicroBrightField, Williston, VT, USA). The densitometric analysis of the acquired images was performed.

### Data analysis and statistics

Concerning the behavioral test data, the differences between the experimental groups were analyzed with two-way ANOVA followed by Bonferroni post-hoc test. Data are expressed as mean ± standard error of the mean (SEM). Statistical analysis of onset and survival rate were detected with Log-rank (Mantel-Cox) test.

Densitometric analysis of Western blot performed with GeneSys software (Singene, UK) and the stereological MNs count data were analyzed using one-way ANOVA followed by Tukey’s multiple for comparisons tests. Data are reported as mean ± SEM. For all statistical analysis and graphs, GraphPad Prism 5 Software was used and significance was accepted at p < 0.05.

## Electronic supplementary material

Supplemental Figures
