# Supplementary material for: Acetylation state of RelA modulated by epigenetic drugs prolongs survival and induces a neuroprotective effect on ALS murine model
Source: Sci Rep. 2018 Aug 27;8:12875. doi: 10.1038/s41598-018-30659-4 (PMC6110772; doi:10.1038/s41598-018-30659-4)
Supplement: Supplementary file 1 — Supplemental Figures [file 41598_2018_30659_MOESM1_ESM.pdf]

Acetylation state of RelA modulated by epigenetic drugs prolongs survival and induces a neuroprotective effect on ALS murine model

Authors Lorenzo Schiaffino[1], Roberta Bonafede [1], Ilaria Scambi[1], Edoardo Parrella[2], Marina Pizzi[2§], Raffaella Mariotti [1§]

Affilations:

[1]Dept. of Neurological, Biomedical and Movement Science; University of Verona; Verona.

[2]Dept. of Molecular and Translational Medicine; University of Brescia; Brescia.

§ Contributed equally

Corresponding Author:
Mariotti R.:
Department of Neurological, Biomedical and Movement Science; University of Verona, School of Medicine, Verona 37134, Italy.
Correspondence and requests for materials should be addressed to Mariotti R. (email: raffaella.mariotti@univr.it).

Supplemental Figure 1

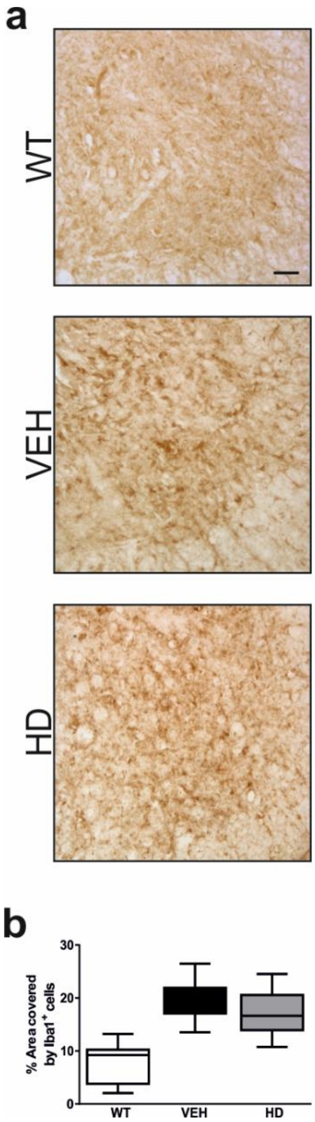

Supplemental Figure 1: The evaluation of microglial activation was performed analyzing the area covered by positive Iba1 cells in the lumbar spinal cord of WT (n=3), VEH (n=3) and HD (n=3). (a) Representative images of lumbar spinal cord of WT, VEH and HD, showed a slight decrease of microglial activation. (b) Graph show the percentage of the area covered by positive Iba1 cells. Despite a slight decrease of the positive Iba1 cells were detected not significant statistical differences were found. Magnification 20x, scale bar 100 μm. Results were analyzed by one-way ANOVA followed by Tukey's multiple comparisons test. Data are expressed as mean±SEM.

Supplemental Figure 2

Figure 4a

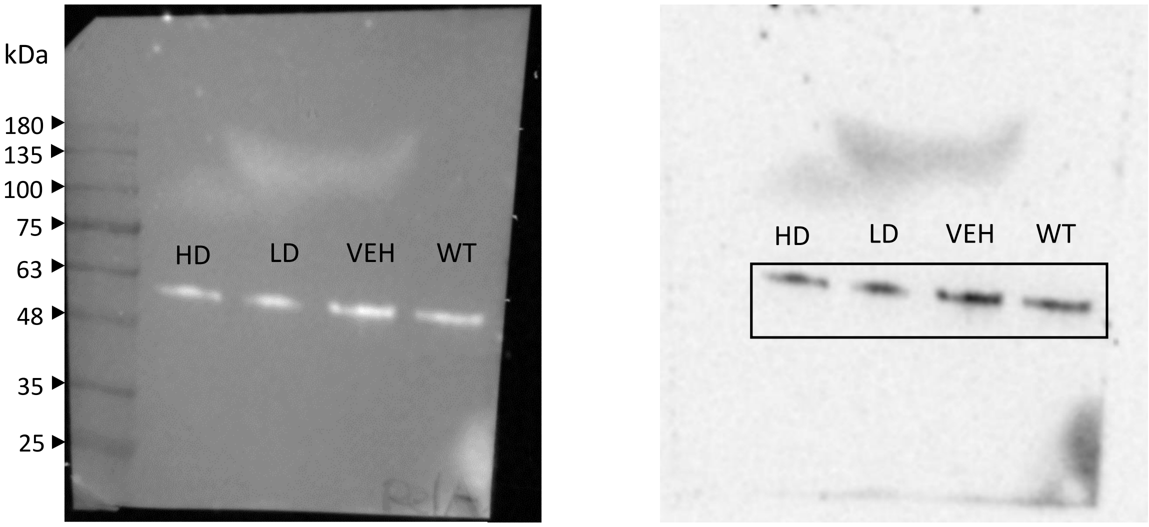

Blot 1 – RelA

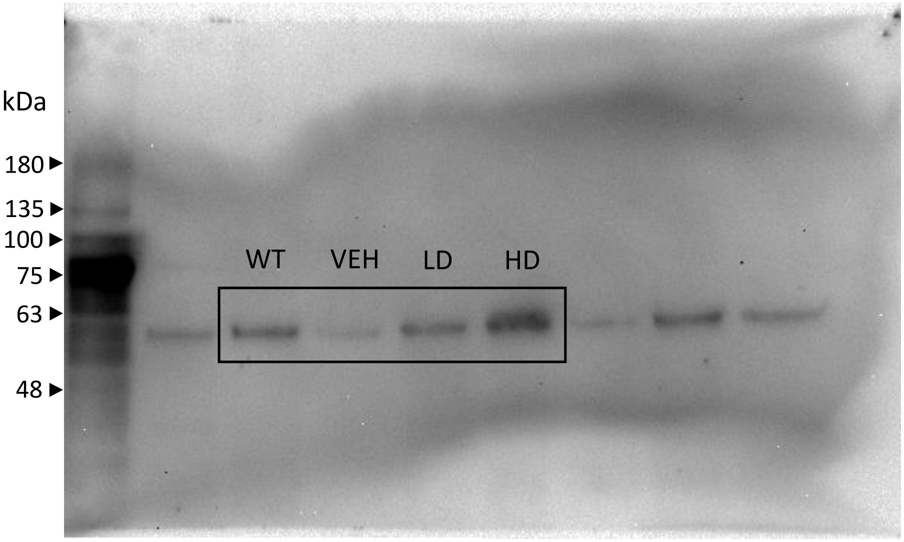

Blot 2 – RelA Ac-K

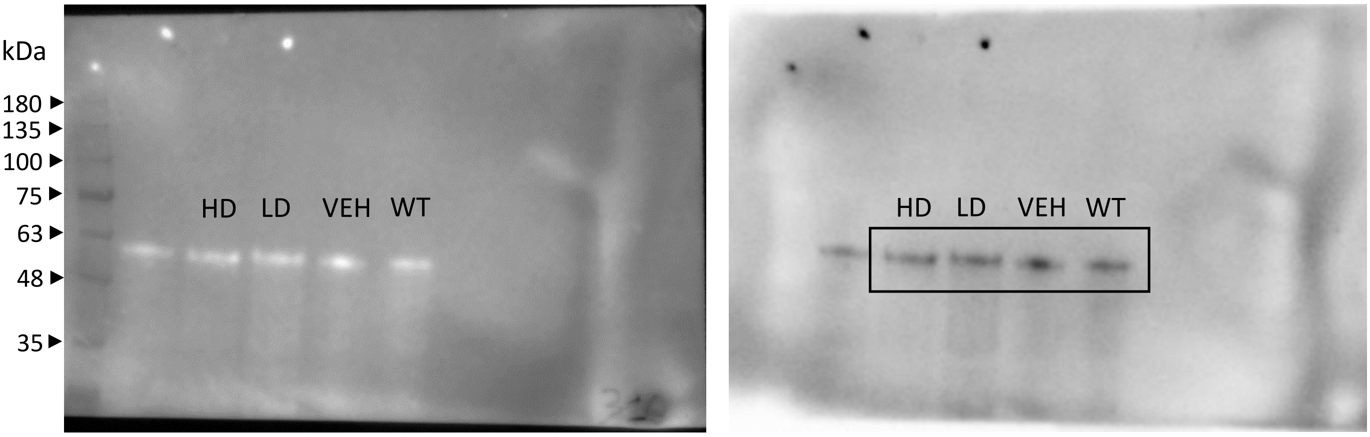

Blot 3 – RelA Ac-K310

Supplemental Figure 2: Immunoprecipitation followed by western blot of RelA subunit of the nuclear fraction isolated from lumbar spinal cord of WT, VEH, LD and HD groups. On the left of *Blot 1 – RelA* and *Blot 3 – RelA Ac-K310* are shown merged blots with the protein molecular weight marker (kDa), on the right are shown the uncropped full length western blots of gels presented in Figure 4a of the main article. The figure *Blot 2 – RelA Ac-K* is displayed with the protein molecular weight marker (kDa). For all blots, the black-lines boxes referred to the cropped parts that are showed in the main article.

Supplemental Figure 3

Figure 6a

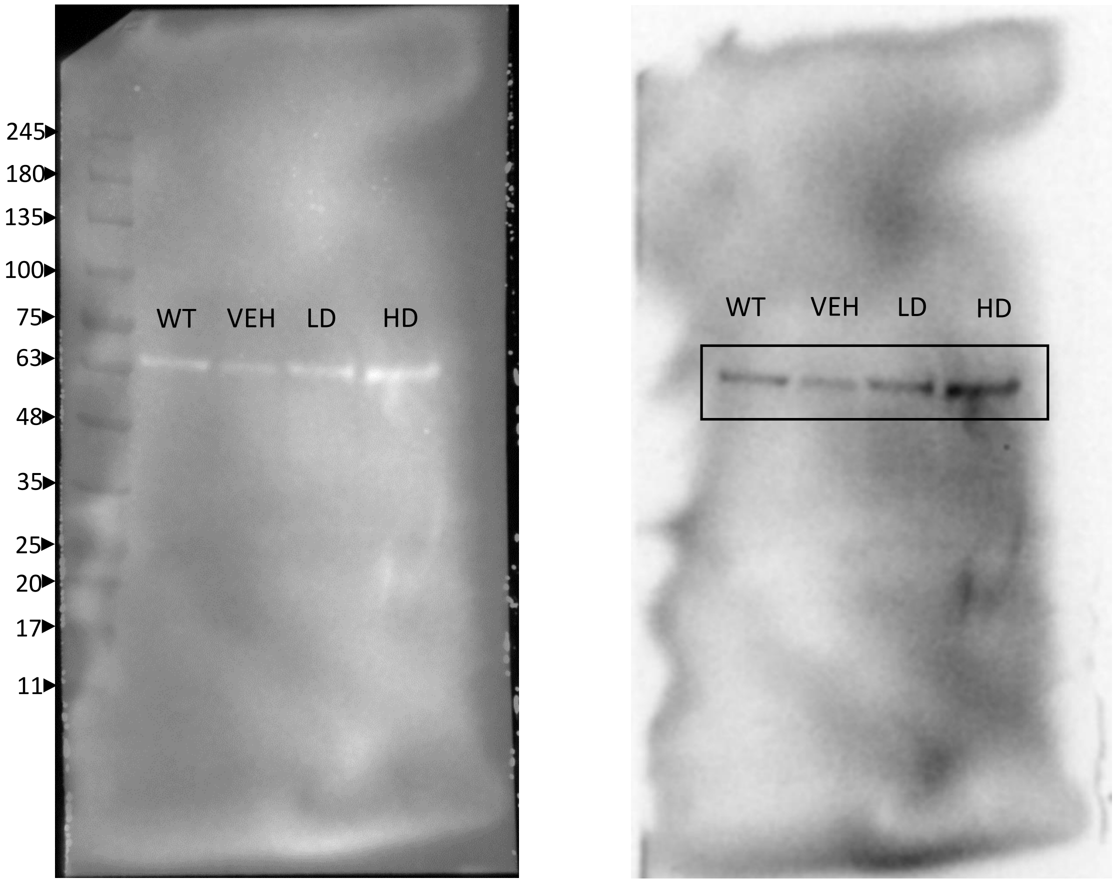

Blot 1 – pAMPK

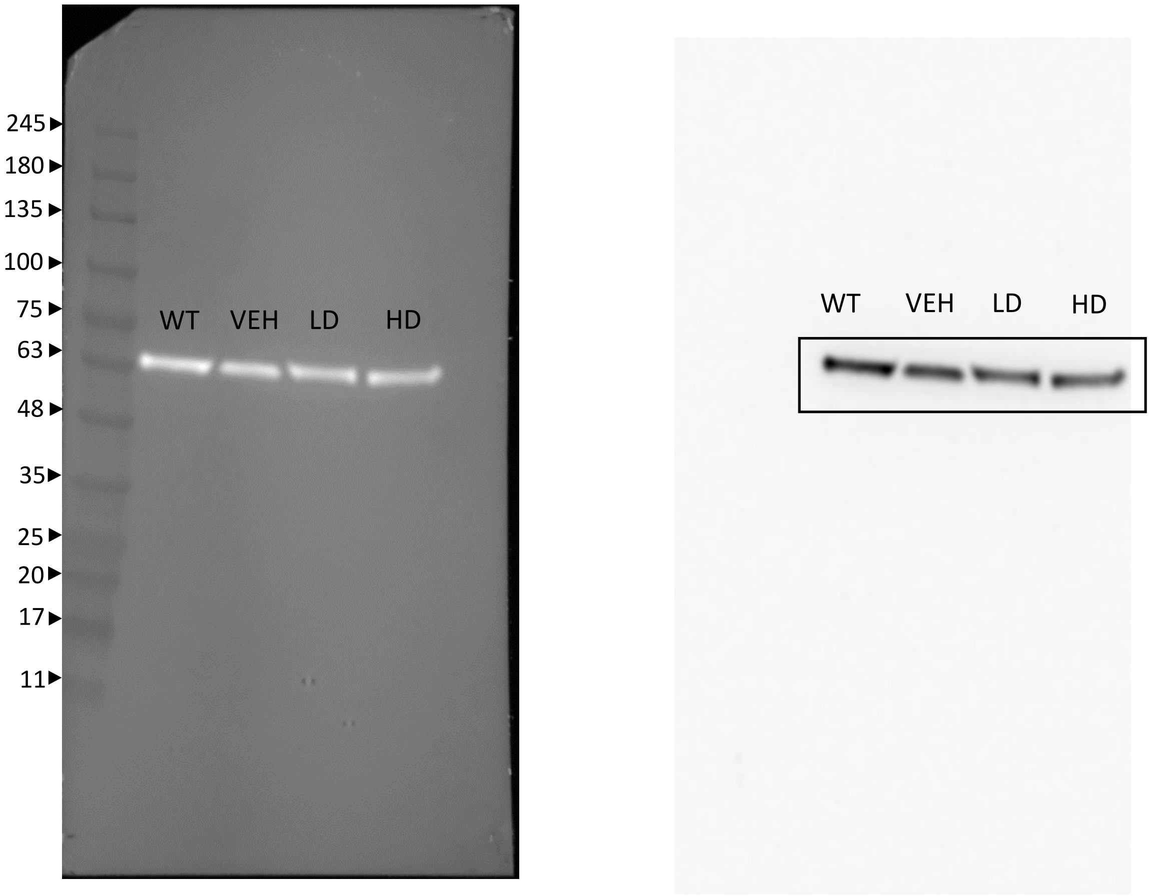

Blot 2 – AMPK

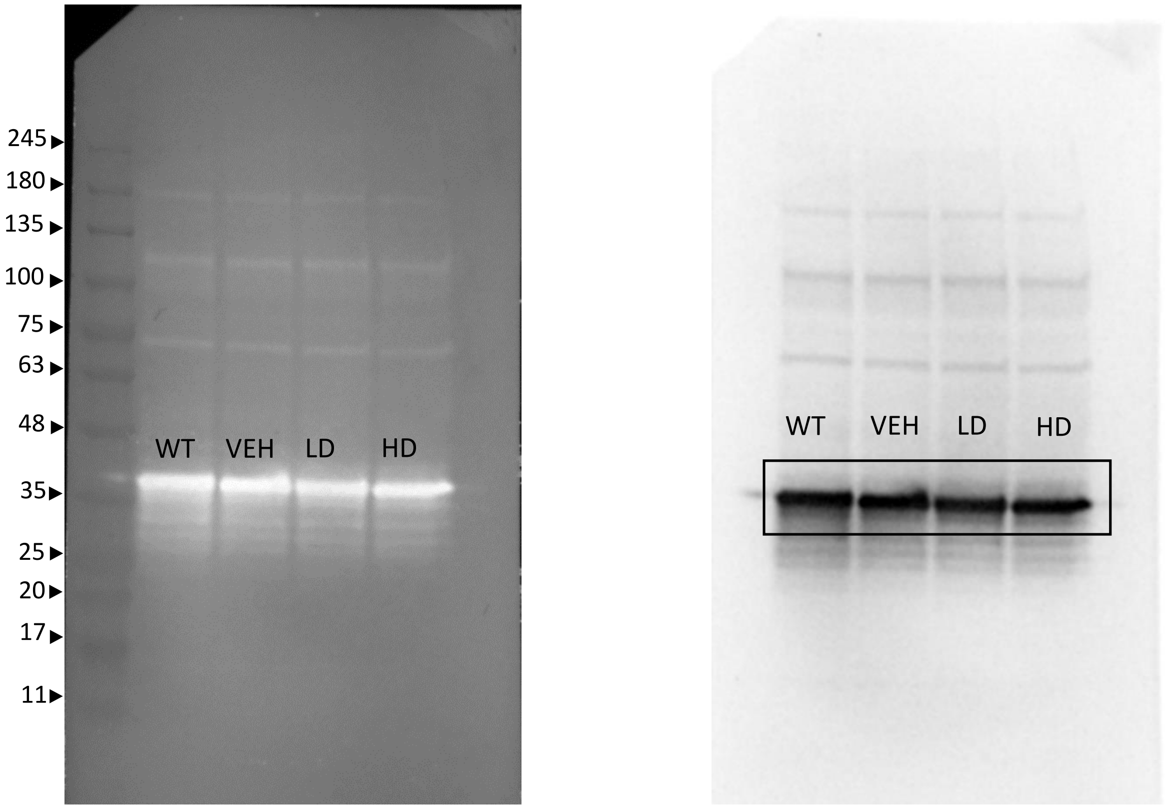

Blot 3 – GAPDH

Supplemental Figure 3: Western blot of cytoplasmic fraction isolated from lumbar spinal cord of WT, VEH, LD and HD groups probed with pAMPK (62kDa), AMPK (62kDa) and GAPDH (37kDa) antibodies. The figure shows the uncropped full length western blots of gels presented in Figure 6a of the main article. The black-lines boxes referred to the cropped parts that are showed in the main article. In the left parts of *Blot 1 – pAMPK*, *Blot 2 – AMPK* and *Blot 3 – GAPDH* are showed merged blots with the protein molecular weight marker (kDa).

Supplemental Figure 4

Figure 7a

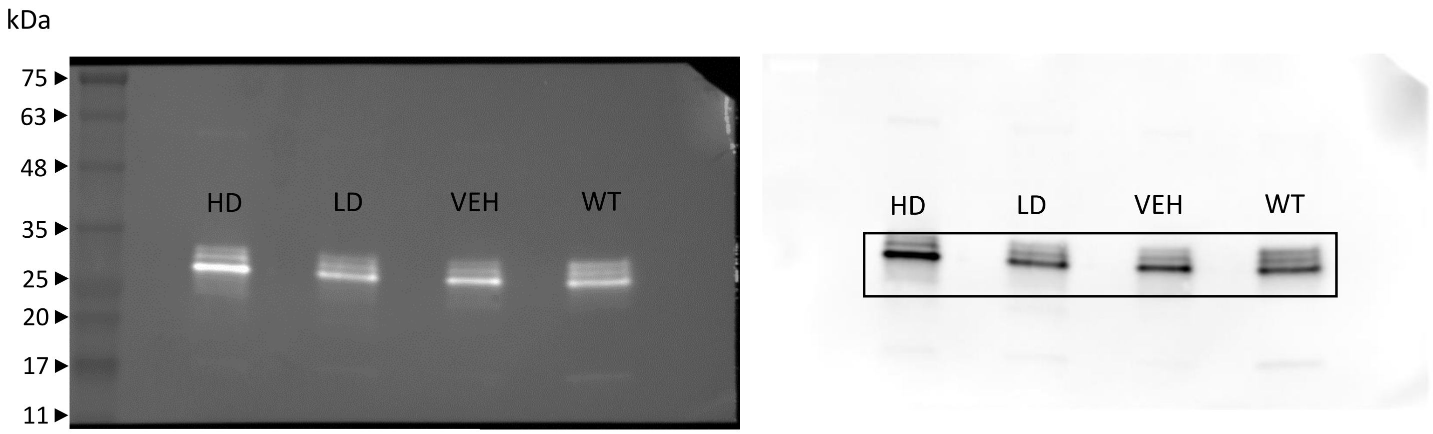

Blot 1 – Bcl-xL

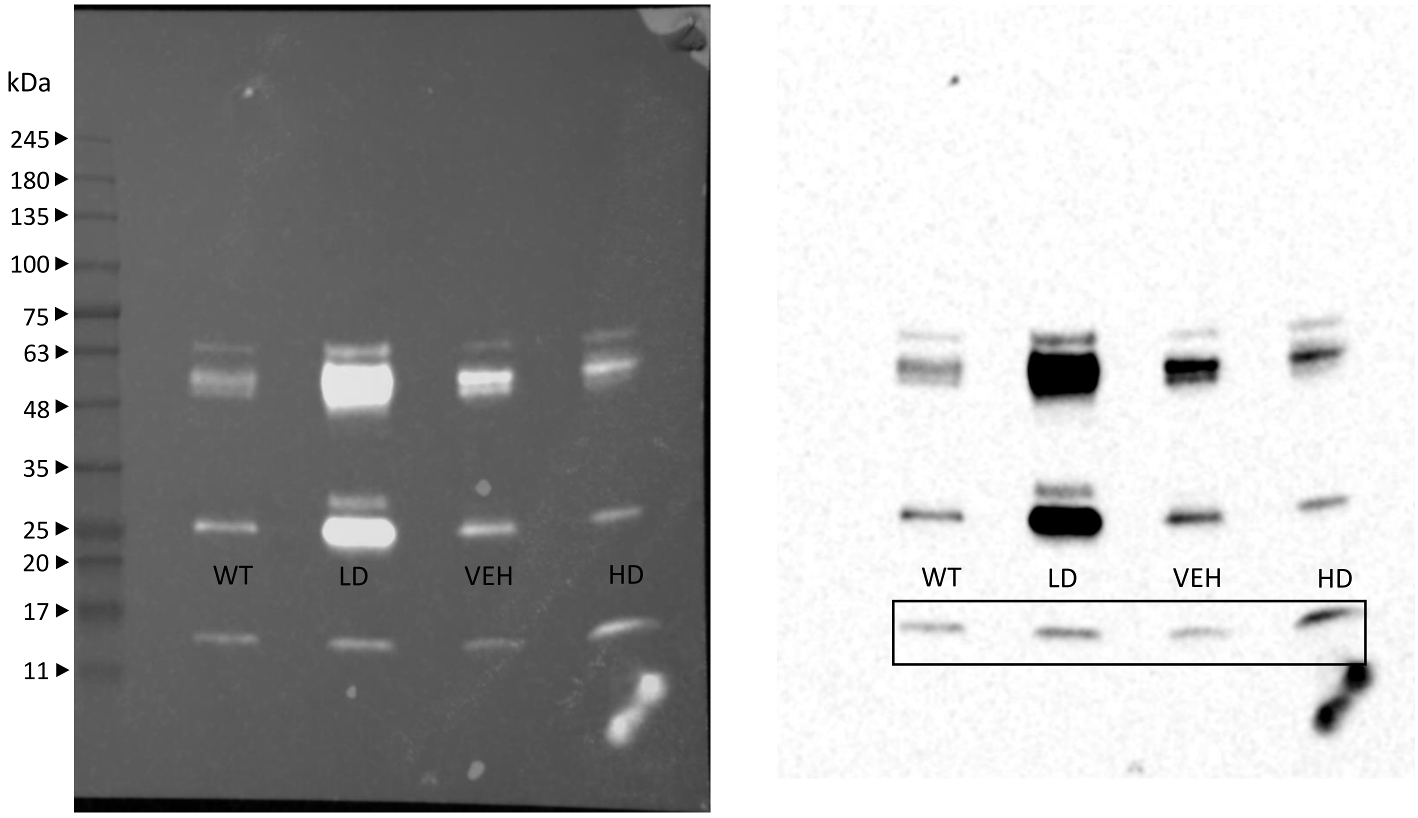

Blot 2 – BDNF

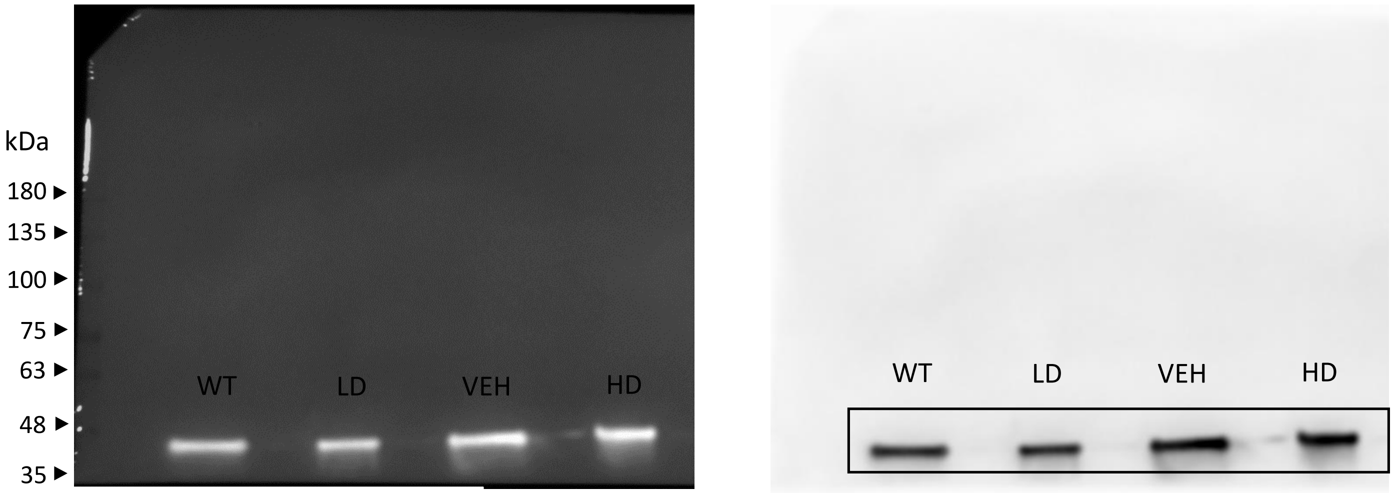

Blot 3 – β-actin

Supplemental Figure 4: Western blot of cytoplasmic fraction isolated from lumbar spinal cord of WT, VEH, LD and HD groups probed with Bcl-xL (30kDa), BDNF (14kDa) and β-actin (45kDa) antibodies. The figure shows the uncropped full length western blots of gels presented in Figure 7a of the main article. The black-lines boxes referred to the cropped parts that are showed in the main article. In the left parts of *Blot 1 – Bcl-xL*, *Blot 2 – BDNF* and *Blot 3 – β-actin* are showed merged blots with the protein molecular weight marker (kDa).
